# Migratory hoverflies orientate north during spring migration

**DOI:** 10.1098/rsbl.2022.0318

**Published:** 2022-10-05

**Authors:** Will L. Hawkes, Scarlett T. Weston, Holly Cook, Toby Doyle, Richard Massy, Eva Jimenez Guri, Rex E. Wotton Jimenez, Karl R. Wotton

**Affiliations:** Centre for Ecology and Conservation, University of Exeter, Penryn Campus, Penryn, Cornwall, UK

**Keywords:** orientation, syrphid, insect migration, spring migration, pollinator

## Abstract

Migratory hoverflies are long-range migrants that, in the Northern Hemisphere, move seasonally to higher latitudes in the spring and lower latitudes in the autumn. The preferred migratory direction of hoverflies in the autumn has been the subject of radar and flight simulator studies, while spring migration has proved to be more difficult to characterize owing to a lack of ground observations. Consequently, the preferred migratory direction during spring has only been inferred from entomological radar studies and patterns of local abundance, and currently lacks ground confirmation. Here, during a springtime arrival of migratory insects onto the Isles of Scilly and mainland Cornwall, UK, we provide ground proof that spring hoverfly migrants have an innate northward preference. Captured migratory hoverflies displayed northward vanishing bearings when released under sunny conditions under both favourable wind and zero-wind conditions. In addition, and unlike autumn migrants, spring individuals were also able to orientate when the sun was obscured. Analysis of winds suggests an origin for insects arriving on the Isles of Scilly as being in western France. These findings of spring migration routes and preferred migration directions are likely to extend to the diverse set of insects found within the western European migratory assemblage.

## 1. Background

Insects migrate in their trillions globally every year to exploit seasonal resources, improve their reproductive success, and/or escape deteriorating habitats [1–4]. Insect movements spread vital ecological roles over large areas, including pollination, pest control, decomposition and nutrient transfer [1,4–6]. Various approaches have been employed to investigate their orientation during migration, including vertical-looking radars to monitor insects at high altitudes, flight simulator experiments to assess orientation in a controlled environment and vanishing bearings to assess butterfly orientation within a natural setting [1,7–12].

Migratory hoverflies can be hugely abundant and are particularly valued as pollinators and predators of crops pests such as aphids [5,6,13,14]. Understanding the movements of these important insects is vital if we are to benefit from their wide-ranging ecological roles. Radar studies in migratory hoverflies have demonstrated seasonally favourable directions of movement, south in autumn and north in spring [7]. In addition, during the autumnal migratory season, migratory hoverflies caught at ground level and flown in a flight simulator have been shown to use a time-compensated sun compass to orientate south but fail to orientate when the sun is obscured [11]. Equivalent ground evidence is currently lacking for spring hoverfly migrants.

To investigate if spring migratory hoverflies have an innate direction preference, and to see if this direction is influenced by the visibility of the sun or wind direction, we undertook a series of experiments to measure the vanishing bearings of captured, pre-reproductive, migratory hoverflies following their arrival into Cornwall and the Isles of Scilly, UK, in mid-June 2022. We predicted that: (i) hoverflies will continue to orientate northwards in a seasonally favourable direction when given a view of the sky with the sun; (ii) they will lose this ability when the sun is obscured; and (iii) they will use favourable winds to aid their flight north.

## 2. Methods

### (a) Migratory insect arrival and capture

**Figure 1.**
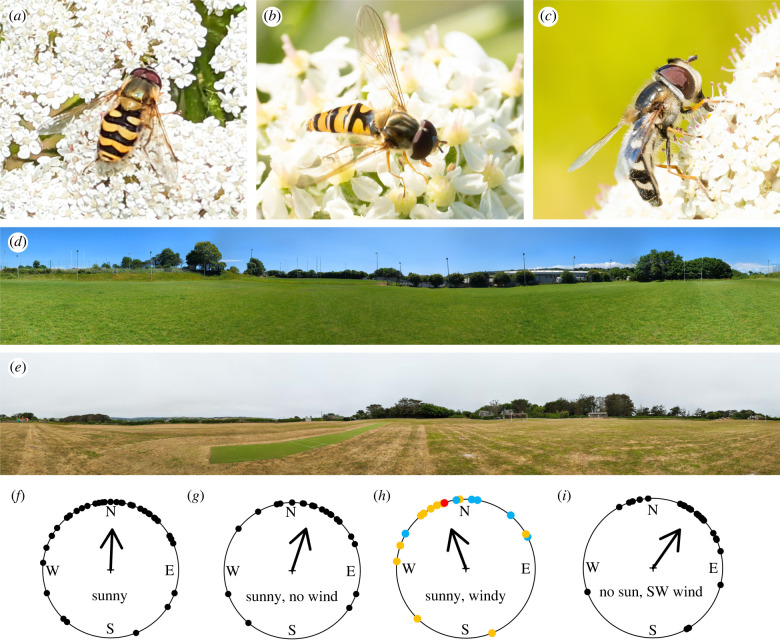
(*a–c*) Photographs of migratory hoverflies taken on the Isles of Scilly prior to capture. (*a*) *Syrphus vitripennis*, (*b*) *Episyrphus balteatus*, (*c*) *Scaeva pyrastri*. (*d*) A 360° panoramic photo of the Penryn site on the day of release in sunny conditions. (*e*) A 360° panoramic photo of the Isles of Scilly study site on the day of release under thick sea mist conditions. (*f*–*i*) Circular histograms of individual vanishing bearings (black dots) and group mean directions (black arrow) with length of the arrow depicting *r* from 0 to 1 at the outer edge of the circle. (*f*) All hoverfly headings while sunny. (*g*) Hoverfly headings with no wind when sunny. (*h*) Hoverfly headings while it was windy and sunny. Yellow dots indicate a south-southeasterly wind, blue dots indicate a westerly wind, red dot indicates an easterly wind. (*i*) Hoverfly headings during a thick sea mist when the sun was obscured, with wind from the southwest.

In 2022, a large influx of migratory insects, consisting of Diptera (the majority being *Syrphus vitripennis*) and Lepidoptera, appeared on the Isles of Scilly and mainland Cornwall between 16 and 21 June. We captured 66 migratory hoverflies and performed vanishing bearing experiments on 16 (*n* = 4) and 17 (*n* = 25) June in the Isles of Scilly. Following these island experiments, we conducted further vanishing bearing experiments in Cornwall on 20 (*n* = 20) and 21 (*n* = 17) June. Five migratory species were caught: *Syrphus vitripennis* (*n* = 45), *Episyrphus balteatus* (*n* = 13), *Scaeva pyrastri* (*n* = 4), *Eristalis tenax* (*n* = 2) and *Syrphus ribesii* (*n* = 2) (figure 1*a–c*). Captured hoverflies were placed into a mesh insect cage and experimented upon within 15 min of capture.

### (b) Vanishing bearings

Two researchers sat east and west, respectively, of the cage of captured hoverflies. Hoverflies were removed from the cage by hand, before being held in the air and released above the researchers' heads. In general, the hoverflies waited longer than 2 s before leaving, while on three occasions, the hoverflies left instantly. On departure, both researchers recorded the heading until the hoverfly was no longer visible (approx. 20 m), and this angle was recorded as the vanishing bearing. Headings were determined using a compass and/or the compass function on a Garmin Instinct Solar watch. Hoverflies were released during three distinct meteorological conditions: (i) sunny with no wind; (ii) sunny with wind (figure 1*d*); and (iii) during a thick sea mist that obscured the sun and with wind (figure 1*e*).

### (c) Location

Experiments took place in two locations: the Isles of Scilly (49.913136 N, −6.3211459 W) and around the Exeter University campus in Penryn, Cornwall (50.166217 N, −5.1192702 W and 50.157906 N, −5.0783014 W). At the sites around the Penryn campus, the experiments were performed in full sunlight between the hours of 11.00 and 16.00 (figure 1*d*). On the Isles of Scilly, the experiments were performed between the hours of 16.00 and 17.00 during a thick sea mist (figure 1*e*). At all locations, experiments were performed on a large open area and the migratory insects (released at least 40 m away from any landscape features above 5 m in height) always had a clear, unobstructed view of the sky.

### (d) Assessing reproductive state of migratory hoverflies

Hoverflies were examined in the field by eye to estimate reproductive state based on the fullness of the abdomen. In addition, the reproductive state of two *S. vitripennis* females was examined under a dissection microscope.

### (e) Meteorological recordings

Meteorological conditions were recorded on location at the same time of the experiments. The on-location measurements were cloud cover (OKTA scale), sun visibility, windspeed (Beaufort scale) and wind direction.

### (f) Visualization of winds

To estimate the potential flight origin, we used Ventusky (www.ventusky.com) to visualize winds at 500 m altitude above sea level on the days leading up to the largest influxes.

### (g) Statistical analysis

Statistical analysis and graphing were carried out in R v. 3.5 [15] using R Studio 1.3 and the circular package [16]. A Rayleigh test was used to analyse the vanishing bearings of all migratory hoverflies and a Mardia–Watson–Wheeler test was used to look for differences in the distributions of wind and insect vanishing bearings, and between the vanishing bearings of *S. vitripennis* females and all other hoverfly migrants. All data are provided in electronic supplementary material, file S1.

## 3. Results

Released when the sun was visible, hoverflies headed almost due north (*θ* = 2.3°, Rayleigh test: *r* = 0.59, *p* < 0.0005, *n* = 41, 95% CI 343.5°–21.1°, figure 1*f*) after an average orientation period of 2.2 s (*n* = 30). Windspeed during recordings when the sun was visible ranged from 0 to 6 m s^−1^ (mean = 1.5 m s^−1^). Under zero-wind conditions, hoverflies headed in a north-northeasterly direction (*θ* = 18.31°, Rayleigh test: *r* = 0.65, *p* < 0.0005, *n* = 24, 95% CI 1.7°–43.6°, figure 1*g*). There was no significant difference between the distribution of these vanishing bearings (Mardia–Watson–Wheeler test: *W* = 35.671, *p* = 0.3). When wind and sun were present simultaneously, the wind direction was either from the west (270°, *n* = 6), the south-southeast (180°–134°, *n* = 11) or the east (90°, *n* = 1). Under these mixed windy conditions, hoverflies headed on average to the north-northwest (*θ* = 340°, Rayleigh test: *r* = 0.5981, *p* = < 0.0005, *n* = 18, figure 1*h*). Owing to low replicates under westerly and easterly wind conditions, we were unable to test for an effect of wind direction on hoverfly vanishing bearings during sunny conditions. However, there was no significant difference between the distribution of south-southeasterly wind headings and vanishing bearings under these conditions (Mardia–Watson–Wheeler test: *W* = 23, *p* = 0.28)*.* Released when the sun was obscured by a thick sea mist, and with a southwesterly wind (238°) blowing at 1.9 m s^−1^, hoverflies headed in a northeasterly direction (*θ* = 35°, Rayleigh test: *r* = 0.67, *p* < 0.0005, *n* = 25, 95% CI 14.7–55.2°, figure 1*i*). There was no significant difference between the distribution of wind headings and vanishing bearings under these conditions (Mardia–Watson–Wheeler test: *W* = 47.732, *p* = 0.3236).

**Figure 2.**
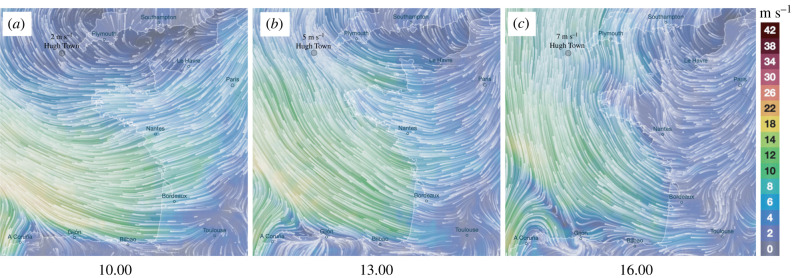
Visualization of wind directions showing the wind headings on 16 June at 10.00, 13.00 and 16.00 at 500 m altitude above sea level. Colour gradients signify wind speeds in m s^−1^. Hugh Town on the Island of St Mary's in the Isles of Scilly is labelled. Source: Ventusky.com.

Mixed sexes and species of migratory hoverflies were analysed in this study. All females examined showed abdomens without significant egg development, and dissections of two female *Syrphus vitripennis* confirmed their pre-reproductive state. Of the 66 hoverflies analysed, 45 were *S. vitripennis* (22 females, 23 males), two were *S. ribesii* (two females), 13 were *E. balteatus* (5 females, 7 males), four were *S. pyrastri* (two females, two males) and two were *E. tenax* (two males). A Mardia–Watson–Wheeler test indicated a lack of significant directional bias by species and sex when comparing the distribution of *S. vitripennis* females with the remainder of the individuals (Mardia–Watson–Wheeler test: *W* = 1.4892, *p* = 0.47). To estimate the potential origin of the migratory hoverflies, we visualized the wind conditions at 500 m above sea level at 10.00, 13.00 and 16.00 on the day preceding the largest influx. These wind conditions suggest an origin for these individuals in France (figure 2).

## 4. Discussion

The utilization of favourable winds has been investigated using radar studies in migratory hoverflies [8]. This study suggested a higher selectivity of favourable wind directions during spring mass migrations than in autumn, and that, unlike in the autumn, springtime hoverflies do not attempt to correct for wind drift and instead orientate themselves with the downwind heading to increase displacement speed [8]. We show here that during a springtime arrival of insect migrants onto the Isles of Scilly and mainland Cornwall, migratory hoverflies orientate and fly in a northerly direction on both sunny and overcast days. Importantly, on sunny days with no wind, hoverflies also headed in a north-northeasterly direction, demonstrating that they are capable of selective orientation during the springtime, rather than simply following the wind. In addition, while the hoverflies were found to fly in a direction not significantly different from the favourable winds, upon release the hoverflies appeared to spend time orientating themselves before leaving, suggesting a period of actively choosing a preferred direction using an internal compass system.

Flight simulator experiments in autumn migrant hoverflies show that they fail to orientate south when the sun is obscured, indicating that other available cues are not used, at least in this experimental set-up [11]. Surprisingly, here we find that spring hoverflies also orientate to the north when the sun is obscured, leaving a question as to the cue being used for orientation under these conditions. One explanation may be that hoverflies are simply orientating in a downwind direction. In support of this, we note that the more easterly vanishing bearings of the hoverflies are consistent with the southwesterly winds during this experiment. Future experiments under different wind conditions or manipulating other potential direction givers are needed to distinguish between these possibilities.

Our analysis of winds suggests that migrants arriving on the Isles of Scilly began their journey in western France, representing a minimum sea crossing of nearly 200 km (figure 2). Radar studies indicate spring hoverflies orientate downwind to increase displacement speed, and show significantly faster speeds than autumn migrants, with an average of 11.2 m s^−1^ [8]. We note very similar speeds from our wind analysis that would suggest this distance could be travelled in 5 h, underlying the importance of warm southerly winds for spring recolonization of northerly latitudes.

The insect migration assemblage is highly diverse, and the findings presented here on orientation behaviour and migratory routes undoubtedly extend to other members of the Syrphidae and perhaps other co-migratory Diptera and Lepidoptera that arrived together in the southwest of the UK. Many of these migratory insects play important ecological roles [5,6,13]; therefore, understanding routes and orientation mechanisms used in the spring provides valuable information to understand and predict migration, and to benefit from and protect the large-scale movements of these insects.

## Data Availability

All data including capture and release date and time, along with the associated meteorological conditions and vanishing bearings, are provided in electronic supplementary material, file S1 in Excel format [17].

## References

[1] Hu G, Lim KS, Horvitz N, Clark SJ, Reynolds DR, Sapir N, Chapman JW. 2016 Mass seasonal bioflows of high-flying insect migrants. Science **354**, 1584-1587. (10.1126/science.aah4379) 28008067

[2] Florio J et al. 2020 Diversity, dynamics, direction, and magnitude of high-altitude migrating insects in the Sahel. Scient. Rep. **10**, 20523. (10.1038/s41598-020-77196-7) PMC7688652 33239619

[3] Dingle H. 2014 Migration: the biology of life on the move. Oxford, UK: Oxford University Press.

[4] Chapman JW, Reynolds DR, Wilson K. 2015 Long-range seasonal migration in insects: mechanisms, evolutionary drivers and ecological consequences. Ecol. Lett. **18**, 287-302. (10.1111/ele.12407) 25611117

[5] Doyle T, Hawkes WLS, Massy R, Powney GD, Menz MHM, Wotton KR. 2020 Pollination by hoverflies in the Anthropocene: pollination by hoverflies. Proc. R. Soc. B **287**, 20200508. (10.1098/rspb.2020.0508) PMC7287354 32429807

[6] Satterfield DA, Sillett TS, Chapman JW, Altizer S, Marra PP. 2020 Seasonal insect migrations: massive, influential, and overlooked. Front. Ecol. Environ. **18**, 335-344. (10.1002/fee.2217)

[7] Wotton KR, Gao B, Menz MHM, Morris RKA, Ball SG, Lim KS Reynolds DR, Hu G, Chapman JW. 2019 Mass seasonal migrations of hoverflies provide extensive pollination and crop protection services. Curr. Biol. **29**, 2167-2173.e5. (10.1016/j.cub.2019.05.036) 31204159

[8] Gao B, Wotton KR, Hawkes WLS, Menz MHM, Reynolds DR, Zhai BP Hu G, Chapman JW. 2020 Adaptive strategies of high-flying migratory hoverflies in response to wind currents. Proc. R. Soc. B **287**, 20200406. (10.1098/rspb.2020.0406) PMC7341907 32486972

[9] Oliveira EG, Srygley RB, Dudley R. 1998 Do neotropical migrant butterflies navigate using a solar compass? J. Exp. Biol. **201**, 3317-3331. (10.1242/jeb.201.24.3317) 9817829

[10] Schmidt-Koenig K. 1979 Directions of migrating monarch butterflies (*Danaus plexippus*; Danaidae; Lepidoptera) in some parts of the eastern United States. Behav. Processes **4**, 73-78. (10.1016/0376-6357(79)90051-2) 24896392

[11] Massy R, Hawkes WLS, Doyle T, Troscianko J, Menz MHM, Roberts NW, Chapman JW, Wotton KR. 2021 Hoverflies use a time-compensated sun compass to orientate during autumn migration. Proc. R. Soc. B **288**, 20211805. (10.1098/rspb.2021.1805) PMC8456149 34547904

[12] Mouritsen H, Frost BJ. 2002 Virtual migration in tethered flying monarch butterflies reveals their orientation mechanisms. Proc. Natl Acad. Sci. USA **99**, 10 162-10 166. (10.1073/pnas.152137299) 12107283 PMC126641

[13] Hawkes WLS et al. 2022 Huge spring migrations of insects from the Middle East to Europe: quantifying the migratory assemblage and ecosystem services. Ecography e06288. (10.1111/ecog.06288)

[14] Aubert J, Aubert JJ, Goeldlin P. 1976 [Twelve years of systematic captures of syrphids (Diptera) at the Bretolet Pass (Valais Alps)]. *J. Swiss Entomol. Soc.* **49,** 115–142. (10.5169/seals-401808) [In French.]

[15] R Core Team. 2020 R: a language and environment for statistical computing. Vienna, Austria: R Foundation for Statistical Computing. See https://www.R-project.org.

[16] Agostinelli C, Lund U. 2017 R package ‘circular’: circular statistics (version 0.4-93). See https://r-forge.r-project.org/projects/circular/.

[17] Hawkes WL, Weston ST, Cook H, Doyle T, Massy R, Guri EJ, Wotton Jimenez RE, Wotton KR. 2022 Migratory hoverflies orientate north during spring migration. Figshare. (10.6084/m9.figshare.c.6214760) PMC9533008 36196552

